# Brain-Gut Axis Modulation of Acupuncture in Functional Dyspepsia: A Preliminary Resting-State fcMRI Study

**DOI:** 10.1155/2015/860463

**Published:** 2015-11-08

**Authors:** Jiliang Fang, Danhong Wang, Qing Zhao, Yang Hong, Yulian Jin, Zhishun Liu, Kehua Zhou, Xianghong Jing, Xiaochun Yu, Ruiqi Pan, Andrew Chang, Hesheng Liu, Bing Zhu

**Affiliations:** ^1^Department of Radiology, Guang An Men Hospital, China Academy of Chinese Medical Sciences, Beijing 100053, China; ^2^Martinos Center for Biomedical Imaging, Massachusetts General Hospital, Harvard Medical School, Charlestown, MA 02129, USA; ^3^Department of Acupuncture and Moxibustion, Guang An Men Hospital, China Academy of Chinese Medical Sciences, Beijing 100053, China; ^4^Department of Health Care Studies, Daemen College, Amherst, NY 14226, USA; ^5^Institute of Acupuncture and Moxibustion, China Academy of Chinese Medical Sciences, Beijing 100700, China; ^6^Department of Chemistry, University of Pennsylvania, Philadelphia, PA 19104, USA

## Abstract

*Objective*. To explore acupuncture effects on brain functional connectivity in patients with functional dyspepsia (FD). *Methods*. Eight patients in an acupuncture treatment group and ten healthy adults in the control group participated in the study. Acupuncture effectiveness was evaluated based on changes of the gastrointestinal symptoms, gastric motility measurements, and gastrin levels and comparisons with the control group when appropriate. To investigate functional connectivity changes related to FD and potential modulation after acupuncture, a set of regions of interest (ROIs) were selected according to previous fMRI reports of acupuncture. *Results*. Patients showed significant improvements of FD signs and symptoms after acupuncture treatments. For all of the ROIs, we identified subportions of the networks showing reduced connectivity in patients with FD. Connectivity between the ROIs and corresponding disease targets showed significant improvement after acupuncture treatment (*P* < 0.05) in all ROIs except for right medial temporal lobe-hippocampus and right inferior parietal lobule. *Conclusion*. Functional connectivity of the brain is changed in patients with FD but approximates that in healthy control after acupuncture treatment. The relief of gastrointestinal signs and symptoms by acupuncture is likely due to the normalization of brain-gut axis associated with FD.

## 1. Introduction

Functional dyspepsia (FD) is a common gastrointestinal disorder without evidence of an organic gastric disease, which affects around 11% to 29.2% of the general population [1]. Despite its high prevalence, characterization of signs and symptoms related to FD has historically been vague and even flawed [2]. The most current definition of FD, as per the Rome III consensus, identifies four characteristic FD symptoms, all assumed to stem from the gastroduodenal region: early satiation, postprandial fullness, epigastric pain, and epigastric burning [3].

In many patients, little can be determined regarding the direct cause of FD besides the general belief that the pathophysiology of FD involves gastrointestinal dysfunction [2]. Recent studies have revealed that the disturbance of the brain-gut axis may be involved in the development of FD [4]. The brain-gut axis refers to a complex, bidirectional communication system between the gastrointestinal tract and the nervous system [5]. The brain-gut axis not only ensures gastrointestinal homeostasis and digestion but also is closely related to affect, motivation, and higher cognitive functions, including intuitive decision [4]. As the importance of the human brain in FD becomes more widely recognized, the evolving treatments of FD began to include drugs targeting at the human brain in addition to typical drugs directly modulating gastrointestinal function [3].

Despite the availability of pharmaceutical interventions, treatments of FD remain unsatisfactory [3]. During the past decades, acupuncture has gained popularity in the management of FD in both China and other Eastern Asian countries. The effectiveness of acupuncture treatment for FD has been supported by some previous studies [6]. For example, Park et al. [7] reported that acupuncture could improve dyspeptic symptoms and quality of life in patients with FD; Ma et al. [8] echoed these results and found acupuncture induced significant symptom relief in as high as 70.69% of patients with FD.

Outside of studies strictly on the therapeutic effectiveness of acupuncture on FD, interest in the general effects of acupuncture stimulation on brain activities of patients with FD has also been explored. Using positron emission tomography-computed tomography (PET-CT) scanning, Zeng et al. [9] identified differences in glycometabolism in certain brain regions of patients with FD as compared to healthy controls and concluded that the AFC (anterior frontal cortex), PCC (posterior cingulate cortex), and caudate tail are areas potentially involved in FD. Acupuncture treatment decreased glycometabolism in the postcentral gyrus and cerebellum and caused simultaneous deactivation of the ACC (anterior cingulate cortex), insula, and hypothalamus in patients with FD [9, 10]. These findings suggest a potential mechanism that acupuncture treatment may improve FD symptoms by acting on particular brain areas.

A powerful approach to analyze large-scale brain networks and their changes after acupuncture modulation is functional connectivity measured by resting-state fMRI. Resting-state functional connectivity measures the spontaneous Blood Oxygenation Level Dependent (BOLD) signal correlation among brain regions and can provide insight into the normally functioning brain as well as functional abnormalities related to brain diseases [11, 12]. Using resting-state fcMRI, here we aimed to identify specific regions showing connectivity changes in patients with FD and further explore whether the connectivity in these brain regions could be normalized after acupuncture treatment.

## 2. Methods

### 2.1. Participants

This is a concomitant study of a clinical trial which included 60 patients with FD, among whom 30 were treated with acupuncture and 30 were treated with sham (nonclassic acupoint) acupuncture [13]. The study was performed at the Department of Acupuncture and the Department of Radiology at Guang An Men Hospital, one of the largest teaching hospitals for traditional Chinese medicine in China. Participants were recruited through advertisements in local newspapers, in posters, and on hospital website. Written informed consent was obtained from each subject before study participation. The study protocol was approved by the Ethics Committee of Guang An Men Hospital.

As part of the original clinical trial study protocol, eight out of the 30 patients in the acupuncture treatment group were randomly selected to participate in the functional MRI study (2 males; mean age 48.38 ± 8.25 yrs). Ten healthy adults were recruited as a control group with age matched to the patient group (3 males; mean age 48.80 ± 8.59 yrs). No significant age or gender difference was found between these two groups (*P* > 0.05). Patients included in the treatment group all met the Rome III diagnostic criteria for FD [14]. Additionally, the following inclusion criteria were applied for both groups: no mental disorders; age between 18 and 70; no pregnancy. All medication related to the gastrointestinal system was stopped one week prior to and during participation, which may include but not be limited to gastric suppression drugs, prokinetics,* H. pylori* eradication agents, and antidepressants.

### 2.2. Acupuncture Protocol

In the patient group, two main acupoints, Zu Sanli (ST36) and Tai Xi (KI3), were used; additional acupoints included Zu Linqi (GB 41), Nei Guan (PC 6), and Shen Men (HT 7). Rationales for the acupoint selection were detailed in a previous publication [13]. Classic acupoints were localized according to the 2008 World Health Organization standards [15]. Needle insertion was perpendicular with a depth of about 25 mm. In order to reach an optimal acupuncture sensation response which is defined as Deqi sensation including soreness, heaviness, fullness, propagation of needling sensation, and/or adjacent muscle twitching [16], moderate acupuncture manipulation of lifting, thrusting, and twirling with a frequency of 120 times/min was performed. Prior to needle removal, the same acupuncture manipulation technique was performed continually to reach one to three times of Deqi sensation (with short interval between Deqi sensations if there was more than one Deqi sensation during the first two minutes); then, the needle was removed. If no Deqi sensation was obtained during the first two minutes, acupuncture needle was left in place for 20 minutes, and one acupuncture manipulation was again applied right before needle removal. Treatment was implemented once every other day, three to four times a week for one month. All acupuncture procedures were performed by the same acupuncturist who had more than six years of clinical experiences with the Huatuo needles (diameter: 0.30 mm, length: 40 mm; Suzhou Medical Appliance Manufactory, Jiangsu, China).

### 2.3. Imaging Data Acquisition and Processing

Brain imaging was performed on a 1.5 Tesla GE Signa MRI system equipped with the standard two channels' birdcage head coil. A spongy cushion was used to minimize the in-scanner head motion. Functional MR images were acquired with the gradient echo EPI sequence (TE 30 ms, TR 2500 ms, matrix 64 × 64, FOV 240 mm, flip angle 90°, slice thickness 3.5 mm, and gap 0.5 mm, 41 slices, paralleled by AC-PC line). Six-minute-long fMRI data were acquired in the ten healthy adults as well as in each patient before and after the one-month acupuncture treatment.

Resting-state fMRI data were processed using the following steps that have been used in previous published studies: (1) slice timing correction (SPM2, Wellcome Department of Cognitive Neurology, London, UK); (2) rigid body correction for head motion with the FSL package [17, 18]; (3) normalization for global mean signal intensity across runs; and (4) low-pass temporal filtering (0.01 Hz–0.08 Hz), head motion regression, and ventricular and white matter signal regression. Whole brain signal regression was also included in the processing stream, which can improve the correction of motion related artifacts [19, 20]. Data of two healthy adults were discarded after quality control due to excessive head motion. All subjects included in the final analyses had met the quality control criterion of slice-based temporal signal-to-noise ratio >100 [21]. The resting-state data were analyzed using region-based correlation analysis, often referred to as functional connectivity MRI (fcMRI) analysis. The present methods extend from Biswal et al. [22] and are described in detail by Wang et al. [23].

To investigate functional connectivity changes related to FD and potential modulation after acupuncture, we selected the following regions of interest (ROIs) according to previous fMRI reports of acupuncture effects on the limbic-paralimbic-neocortical network [24, 25]. These ROIs included pregenual ACC, sub-ACC, MTL-hippocampus, IPL, and anterior insula. Seed regions were defined as spheres with 8 mm radius.

### 2.4. Outcome Assessment

Acupuncture effectiveness on FD was evaluated based on subjective and objective outcomes [13]. In brief, subjective outcomes included dyspeptic symptoms, quality of life, and mental status; objective outcomes included fasting serum gastrin concentration, height of gastric fluid retention, and frequency and propagation velocity of gastric slow wave measured by the barium swallow test. Subjective outcomes were measured in patients with FD before and after one month of acupuncture treatment; objective outcomes were measured in healthy adults as well as in patients with FD before and after one month of acupuncture treatment.

The Chinese version Nepean Dyspepsia Index (NDI) was used to measure both dyspeptic symptom scores and impairment of the dyspepsia-specific health-related QOL (H-QOL). NDI is dyspepsia impact scale developed by Talley et al. [26]. Its Chinese translation version was reported to have high reliability and validity [27]. In the present study, we selected the four cardinal dyspeptic symptoms and their corresponding assessment as reported in the NDI. The score for each symptom in the checklist of cardinal dyspeptic symptoms was calculated by adding its corresponding frequency, severity, and level of discomfort; dyspeptic symptom sum score (DSSS) is the sum score of the four symptoms in the checklist. Quality of life was measured by the short-form 36 (SF-36) questionnaires [28]. Mental status of patients was evaluated via Zung Self-Rating Depression Scale (SDS) [29] and Self-Rating Anxiety Scale (SAS) [30].

A fasting venous blood sample was drawn from the basilic vein prior to breakfast early in the morning. About three milliliters of the blood sample was sent to Peking Union Medical College Hospital for measurement of serum gastrin levels. Meanwhile, the X-ray gastric barium meal dynamic radiography was performed. The participants were given 120 mL 80% (w/v) barium sulfate suspension meal (Qingdao Dongfeng Chemical Co. Ltd., Shandong, China). Participants were then placed in a supine position with trunk rotated. Using the digital X-ray machine (Prestige, GE, USA), gastric mucosa was observed; then, a Chinese coin of fifty cents was placed on top of the skin over the stomach of the participant, and gastric motions around the gastric antrum were video recorded for one minute while the participant was in a standing position. Frequency of gastric slow wave was directly counted as the number of waves that passed through the gastric antrum in one minute. Propagation velocity of gastric slow wave was assessed by the time interval between two consecutive waves that passed through the gastric antrum. Heights of gastric fluid retention were measured in captured pictures and calculated into actual values.

### 2.5. Statistical Analysis for FD Symptoms, Gastrin, and Gastric Motility

The statistical analyses were performed by two independent statisticians who were blind to the treatments and study protocol. Statistical Analysis System (SAS), version 6.12, was used and a significance level was set at *α* < 0.05.

Wilcoxon signed-rank test was used to explore differences in subjective scores and objective measurements before and after acupuncture treatment in patients with FD. Wilcoxon rank-sum test was used to compare objective measurements in patients with FD and healthy volunteers. All quantitative data including subjective scores were expressed with mean ± SD.

## 3. Results

### 3.1. Subjective Outcomes

After one-month acupuncture treatment, patients with FD showed significant improvements in each individual symptom score, dyspeptic symptom sum score, and SDS and SAS scores (see Table 1). The improvement rates vary from 29% to 100% (*P* < 0.05 for all comparisons); the quality of life in patients with FD was significantly improved with a 40% increase in SF-36 scores (Table 1).

### 3.2. Objective Outcomes

Before treatment, patients with FD demonstrated lower levels of fasting serum gastrin concentration and frequency and propagation velocity of gastric slow wave than healthy adults (*P* < 0.05 for all comparisons). After one-month acupuncture treatment, no difference in these measurements was found between the patient and healthy groups (*P* > 0.05 for all measurements). Before acupuncture treatment, six patients had gastric fluid retention with a mean height of 44.35 mm; after the treatment, no patient demonstrated measurable gastric fluid retention (see Table 2).

### 3.3. The Changes of Functional Connectivity Associated with FD

To explore potential functional connectivity changes associated with FD and the modulation effect of acupuncture treatment, we computed the functional connectivity maps using ten predefined ROIs in the default network and attentional network, including pregenual ACC, subgenual ACC (sub-ACC), medial temporal lobe-hippocampus (MTL-Hippocampus), inferior parietal lobule (IPL), and anterior insula (Anti-Insula) in each hemisphere. Two additional ROIs were defined in the primary visual cortex for a control analysis (Table 3).

**Figure 1:**
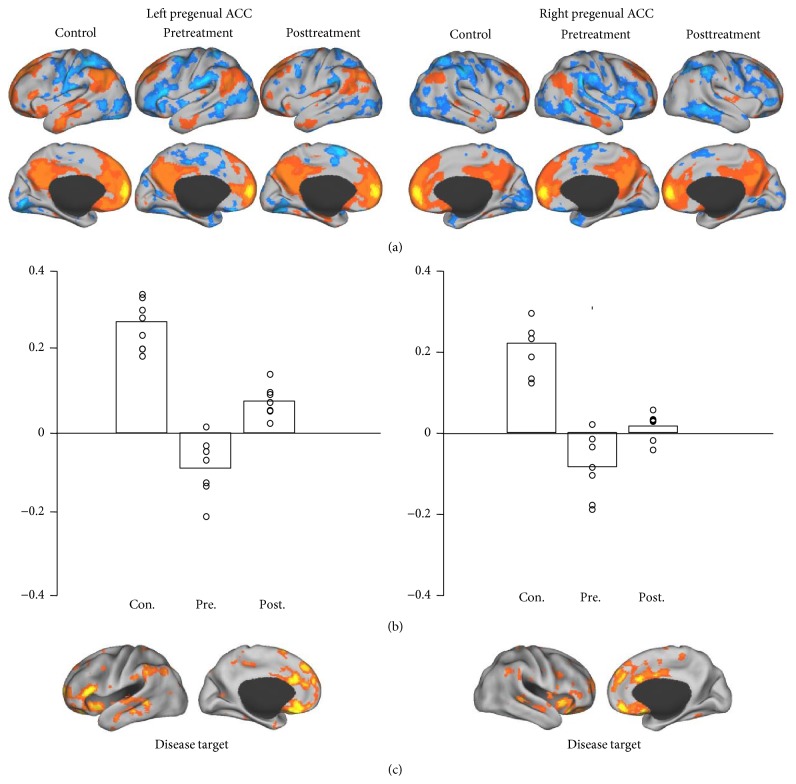
Functional connectivity maps of controls and pretreatment and posttreatment groups were computed based on the pregenual ACC ROI (a). The left pregenual ACC seed region was connected with insula, dorsal lateral prefrontal cortex (dLPFC), medial prefrontal cortex, supramarginal gyrus, and superior and middle temporal gyri. The right pregenual ACC seed region was connected with insula, medial prefrontal cortex, middle cingulate gyrus, lateral prefrontal cortex, supramarginal gyrus, and middle temporal gyrus. Reduced connectivity was found in pretreatment group compared with healthy adults. These brain regions, termed disease targets, represent the regions associated with the FD disease (c). After treatment, connectivity was enhanced with the tendency to approximate the healthy controls. The connectivity values between pregenual ACC and the disease target were plotted for controls and pretreatment and posttreatment groups (b).

**Figure 2 fig2:**
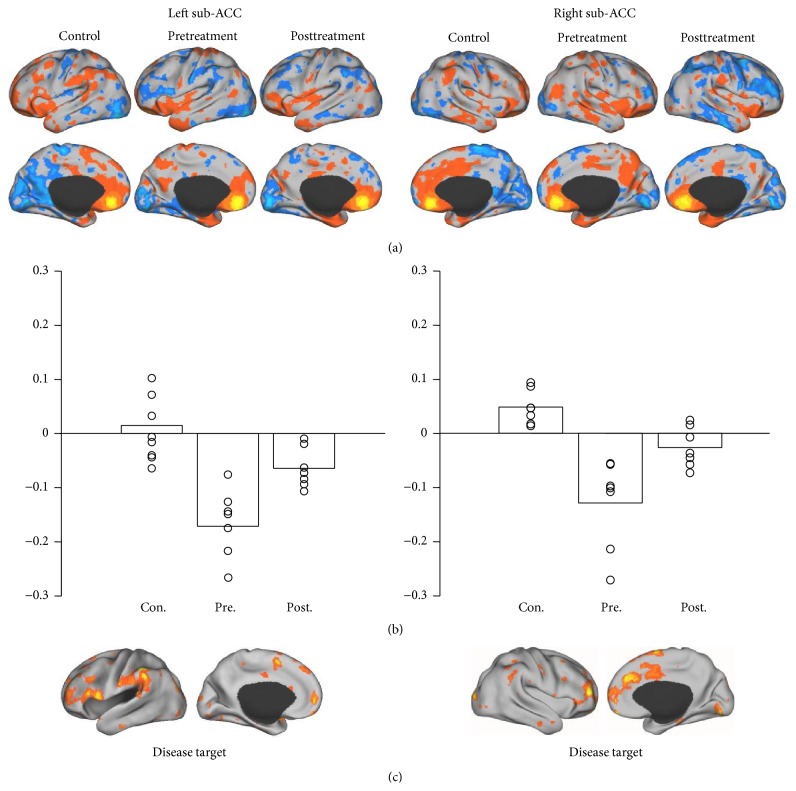
Regions connected with the sub-ACC ROI. The regions strongly correlated with left sub-ACC included dorsal lateral prefrontal cortex, supramarginal gyrus, medial prefrontal cortex, and medial supplementary motor area. The right sub-ACC correlated regions included medial prefrontal cortex, middle cingulate gyrus, and frontal pole.

**Figure 3 fig3:**
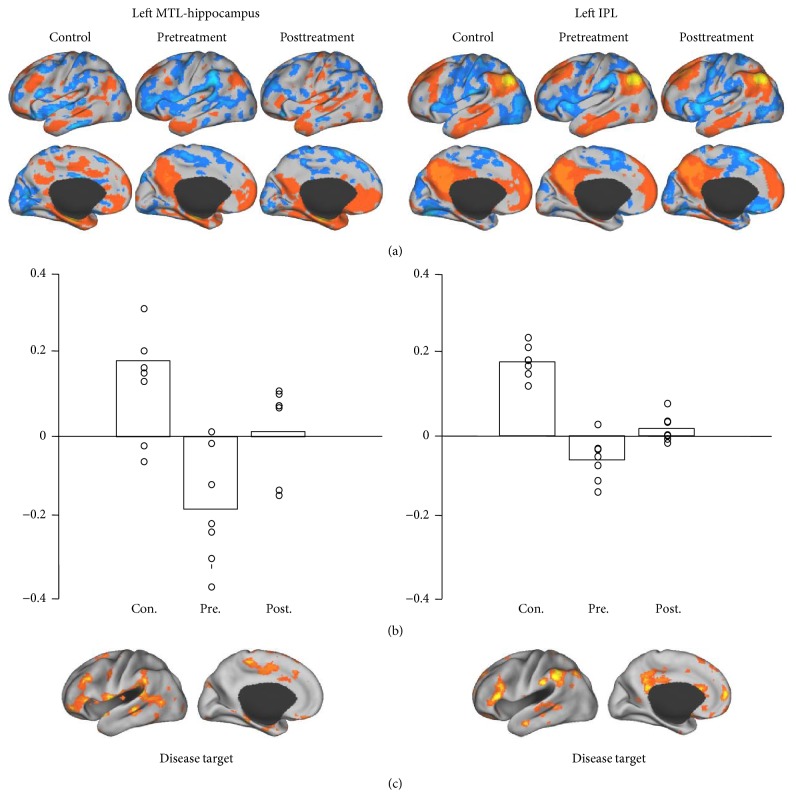
Seeds were placed in MTL-hippocampus and IPL. Treatment-related improvement only showed in the left hemisphere. The left MTL-hippocampus seed regions were correlated with lateral prefrontal cortex, orbital gyrus, and middle and posterior cingulate gyrus. The regions strongly correlated with left IPL seed included inferior lateral prefrontal cortex, supramarginal gyrus, posterior cingulate anterior cingulate cortex, and medial prefrontal cortex.

**Figure 4:**
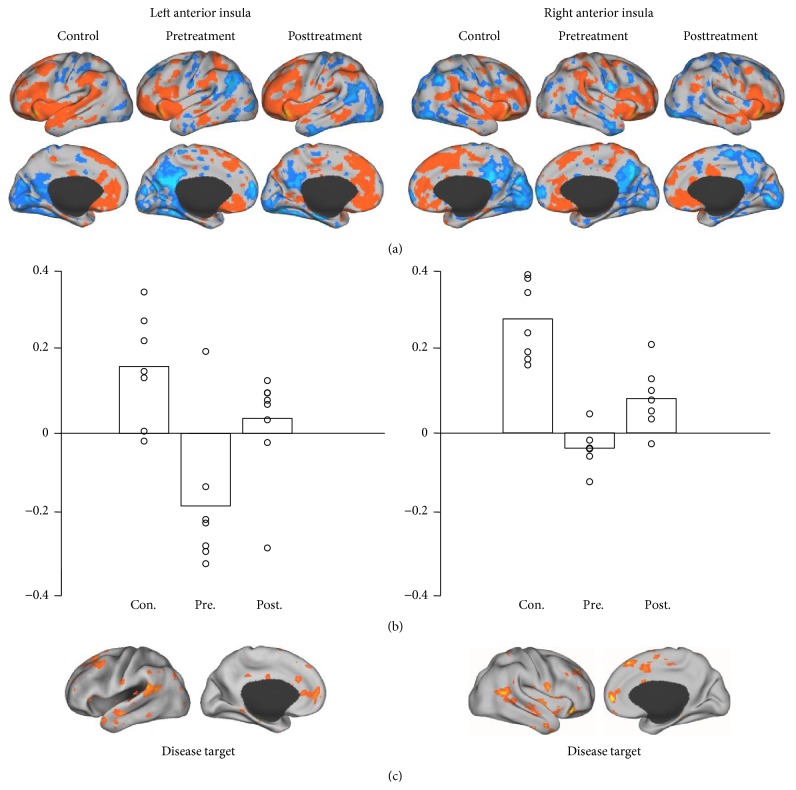
A seed was placed in anterior insula. The left seed region correlated with anterior cingulate cortex, supplementary motor area, supramarginal gyrus, and superior and middle temporal gyri. The right seed correlated with medial prefrontal cortex, medial supplementary motor area, and middle temporal gyrus.

In each of the three groups (control and pre- and posttreatment groups), we identified the regions demonstrating strong functional connectivity to the ROIs ((a) in Figures 1 [Fig fig2] [Fig fig3]–4). For each ROI, we then identified the regions showing strong connectivity difference between the healthy control group and the pretreatment group (mean group difference *Z* > 0.1). These regions were considered as the “disease targets,” representing the regions whose connectivity with the ROIs was affected by FD ((c) in Figures 1–4). We then examined if the connectivity between each ROI and its corresponding “disease target” can be normalized by the acupuncture treatment. The connectivity values between each ROI and its corresponding disease target were plotted ((b) in Figures 1–4) and compared between the pre- and posttreatment groups.

For all of the ROIs in the default and attentional network, we could identify subportions of the networks showing reduced connectivity in patients with FD (see (c) in Figures 1–4). Connectivity between the ROIs and corresponding disease targets showed significant improvement after acupuncture treatment (*P* < 0.05, Wilcoxon paired nonparametric test) in all ROIs except for right MTL-hippocampus and right IPL.

**Figure 5:**
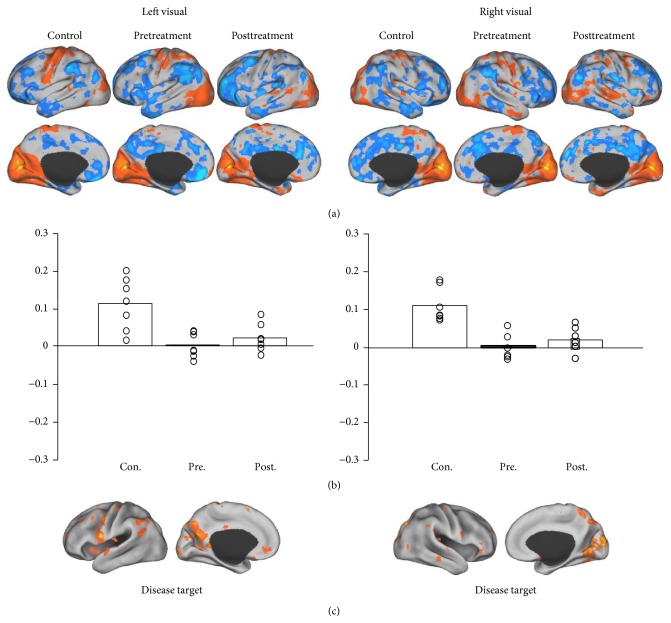
As a control analysis, two seeds were placed in the primary visual cortex. Patients with FD showed reduced connectivity between some sensorimotor regions and visual cortex. However, there was no improvement after acupuncture treatment.

As a control analysis, we placed ROIs in the primary visual cortex because it is known that acupuncture does not have significant effect on the visual system in FD treatment [9, 10, 31]. The goal of this control analysis is to test whether the posttreatment improvement was due to any data quality difference between groups. We found some regions showing reduced connectivity to visual ROIs in patients with FD. However, the connectivity was not changed after acupuncture treatment (Figure 5).

Of note, no significant correlation was found between the connectivity changes in these brain regions with the changes of patients' clinical symptoms, gastric motility scores, and serum gastrin concentration. This is partially due to the limited number of subjects and potentially highly complicated neural mechanisms responsible for the symptoms.

## 4. Discussion

### 4.1. Effect of Acupuncture on Functional Dyspepsia Symptoms and Signs

The history of acupuncture treatment for digestive problems like FD dates back thousands of years; the beneficial effects of acupuncture for FD have been noted by generations of practitioners and are supported by modern research [6–8]. Through randomized controlled trials, previous studies reported that patients with FD experienced improvements in FD symptoms after classic acupuncture treatment as compared with acupuncture at nondefined points after two to four weeks of treatment [7, 8, 10]. In another study, Lima et al. [32] found that combining drug therapy and classic acupuncture treatment was superior to combining drug therapy and acupuncture at nondefined acupoints. The alleviation of symptoms reported in the present study is consistent with the previous studies on the effects of acupuncture [6–8], supporting the efficacy of acupuncture treatment in gastrointestinal disorders like FD.

In the present study, acupuncture was found to increase serum fasting gastrin level in patients with FD toward normal values of healthy controls. The result was consistent with previous acupuncture studies [33, 34]. Increased secretion of serum fasting gastrin has been reported by Chang et al. [33] using acupuncture at ST36 and Zhang [34] using acupuncture at simultaneous front-Shu and back-Mu acupoints. Nonetheless, the enhanced secretion of gastrin and brain effects of acupuncture as demonstrated in the present study suggested that the relief of FD symptoms by acupuncture may be due to its modulation of the brain-gut axis.

### 4.2. Altered Brain Functional Connectivity in the Brain-Gut Axis of FD and the Acupuncture Effects

The involvement of the brain-gut axis in gastrointestinal system homeostasis is widely supported with understandings of the enteric nervous system and the neurobiological pathways involved in its modulation. Previous studies have demonstrated the involvement of the brain-gut axis in digestive disorders similar to FD. For example, an investigation of irritable bowel syndrome (IBS) using fMRI reported that psychosocial stress and activation of three brain areas (midcingulate cortex, somatosensory cortex, and prefrontal area) were associated with worsened IBS symptoms [35]. In the same study, researchers also found that improvements in IBS symptoms were associated with decreased activation of these particular brain regions [35]. Additionally, Jonsson et al. [36] reported that anxiety-provoking interview increased gastrin levels in patients with FD. These findings suggest that emotional and psychological changes may affect functions of the digestive system. One of the understandings in such a phenomenon could be due to the close relationship between the enteric nervous system (ENS) and the central nervous system (CNS). In a review article, Natale et al. [37] summarized recent research studies and concluded that misfolded protein aggregates in both the ENS and CNS in neurodegenerative diseases can theoretically impact the gastrointestinal functions in patients with neurodegenerative disorders. Our previous studies demonstrated that acupuncture could modulate the limbic-paralimbic-neocortical network and limbicprefrontal regions that are essential for emotional control and cognition [24, 25]. Thus, it is possible that acupuncture modulates specific brain cognitive networks of the brain-gut axis in patients with FD.

Consistent with the hypothesis, acupuncture has been found to affect brain activities in patients with FD [9, 10, 31]. Using PET-CT, researchers identified various brain regions related to possible abnormal brain activities and the modulatory effects of acupuncture on some of these brain regions [9, 10]. The AFC, PCC, and caudate tail areas were found to be potentially involved in FD [9, 10]. While PET-CT may be able to detect intrinsic activities of the human brain based on metabolic activities [9], fMRI provides another tool for the exploration of central effects of acupuncture on FD. In a fMRI study, Liu et al. [31] found positive correlations between the activity in dorsomedial prefrontal cortex (dmPFC) and pregenual anterior cingulate cortex (pACC) and the symptom severity, as well as positive correlations between the activity in middle cingulate cortex (MCC), OFC, insula, temporal pole (TP), and FD duration.

Using resting-state fcMRI, the present study investigated functional connectivity abnormalities in FD and the potential effect of acupuncture on connectivity. We identified a set of brain regions that demonstrated reduced functional connectivity in patients with FD, including regions in the default and attentional networks that are suggested to be responsible for symptoms related to depression and psychosis [24, 25, 38]. Our results thus provide evidence for the brain-gut axis theory connecting functions of the gastrointestinal system with the central nervous system.

Importantly, we found that connectivity was enhanced with the tendency to approximate the healthy controls after acupuncture treatment. Acupuncture tended to normalize changes of brain activities in the aforementioned brain areas, such as connectivity between pregenual ACC and medial precuneus, bilateral frontal pole, orbital gyrus and medial gyrus of the frontal cortex (BA10, BA11), and bilateral middle gyrus of frontal cortex (BA43, BA45, and the middle lower part of BA4) in patients with FD; these results are partially consistent with previous research studies which demonstrated positive effects of acupuncture on patients with FD. Our results add further credence to acupuncture's role in normalizing the brain-gut axis related to FD.

Certainly, our sample size of eight patients in this study was relatively small, in part due to difficulties in obtaining fcMRI scan data for a large number of patients before and after acupuncture. Nonetheless, we believe that the subjective and objective outcome results, as well as the imaging findings, strongly support the fact that acupuncture can effectively treat FD by modulating neural activity in specific cognitive brain regions. Further investigation of functional connectivity and the brain's role in the pathophysiology should be pursued with a larger number of subjects.

## 5. Conclusions

Functional connectivity of the brain is changed in patients with FD but approximates that in healthy control after acupuncture treatment. The relief of gastrointestinal signs and symptoms in FD by acupuncture is likely due to the normalization of brain-gut axis associated with FD.

## Figures and Tables

**Table 1:** Subjective measurements (symptoms scores) of 8 patients with FD (mean ± SD).

Measurements	*N*	Baseline	After	Difference	Improvement (%)	*P* value
PF	8	9.75 ± 1.75	1.63 ± 2.13	8.13 ± 2.03	83.38%	<0.0001
ES	8	9.75 ± 2.71	0.75 ± 2.12	9.00 ± 2.83	92.31%	<0.0001
EP	7	6.71 ± 2.43	0.71 ± 1.25	6.00 ± 1.91	89.42%	<0.0001
EBS	5	6.60 ± 2.19	0.00 ± 0.00	6.60 ± 2.19	100.00%	<0.0001
DSSS	8	29.5 ± 7.25	3.00 ± 4.28	26.5 ± 5.76	89.83%	0.0002
SF-36	8	50.77 ± 16.22	71.20 ± 14.78	20.43 ± 22.39	40.24%	0.0364
SDS	8	61.88 ± 11.68	44.00 ± 8.64	17.88 ± 12.88	28.89%	0.0057
SAS	8	57.88 ± 13.00	41.00 ± 6.39	16.88 ± 13.87	29.16%	0.0108

PF: postprandial discomfort; ES: early satiety; EP: epigastric pain; EBS: epigastric burning sensation; DSSS: dyspeptic symptom sum score; SF-36: short-form 36 questionnaire; SDS: Self-Rating Depression Scale; SAS: Self-Rating Anxiety Scale.

**Table 2:** Objective measurements in patients with functional dyspepsia and healthy adults.

Items	A: baseline (*n* = 8)	B: after treatment (*n* = 8)	C: healthy adults (*n* = 10)	*P* values		
				A versus B	A versus C	B versus C
Gastrin (pg/ml)	25.93 ± 5.90	44.4 ± 6.26	47.65 ± 20.21	0.0002	0.0081	0.6401
FGSW (n/min)	2.49 ± 0.64	3.11 ± 0.14	3.11 ± 0.13	0.0078	0.0008	1.0000
PVGSW (s)	24.25 ± 4.95	19.75 ± 2.05	19.41 ± 0.93	0.0180	0.0279	0.6713

FGSW: frequency of gastric slow wave; PVGSW: propagation velocity of gastric slow wave.

Note: propagation velocity of gastric slow wave was assessed by the time interval between two consecutive waves that passed through the gastric antrum.

**Table 3:** Locations of seed regions used to quantify effectiveness of acupuncture.

Seed region	MNI coordinates
Left/right Anti-Insula	−32, 24, −8/32, 24, −8
Left/right pACC	−3, 48, 7/3, 48, 7
Left/right sub-ACC	−10, 32, −6/10, 32, −6
Left/right IPL	−46, −68, 36/50, −62, 32
Left/right MTL-hipp	−26, −20, −18/26, −20, −18
Left/right vision	−10, −82, 8/10, 82, 8

## References

[1] Mahadeva S., Goh K.-L. (2006). Epidemiology of functional dyspepsia: a global perspective. *World Journal of Gastroenterology*.

[2] Geeraerts B., Tack J. (2008). Functional dyspepsia: past, present, and future. *Journal of Gastroenterology*.

[3] Camilleri M., Di Lorenzo C. (2012). Brain-gut axis: from basic understanding to treatment of IBS and related disorders. *Journal of Pediatric Gastroenterology and Nutrition*.

[4] Mayer E. A. (2011). Gut feelings: the emerging biology of gut-brain communication. *Nature Reviews Neuroscience*.

[5] Camilleri M., Stanghellini V. (2013). Current management strategies and emerging treatments for functional dyspepsia. *Nature Reviews Gastroenterology and Hepatology*.

[6] Lan L., Zeng F., Liu G. J. (2014). Acupuncture for functional dyspepsia. *Cochrane Database of Systematic Reviews*.

[7] Park Y.-C., Kang W., Choi S.-M., Son C.-G. (2009). Evaluation of manual acupuncture at classical and nondefined points for treatment of functional dyspepsia: a randomized-controlled trial. *Journal of Alternative and Complementary Medicine*.

[8] Ma T. T., Yu S. Y., Li Y. (2012). Randomised clinical trial: an assessment of acupuncture on specific meridian or specific acupoint vs. sham acupuncture for treating functional dyspepsia. *Alimentary Pharmacology and Therapeutics*.

[9] Zeng F., Song W.-Z., Liu X.-G. (2009). Brain areas involved in acupuncture treatment on functional dyspepsia patients: a PET-CT study. *Neuroscience Letters*.

[10] Zeng F., Qin W., Ma T. (2012). Influence of acupuncture treatment on cerebral activity in functional dyspepsia patients and its relationship with efficacy. *The American Journal of Gastroenterology*.

[11] Fox M. D., Greicius M. (2010). Clinical applications of resting state functional connectivity. *Frontiers in Systems Neuroscience*.

[12] Fox M. D., Raichle M. E. (2007). Spontaneous fluctuations in brain activity observed with functional magnetic resonance imaging. *Nature Reviews Neuroscience*.

[13] Jin Y., Zhao Q., Zhou K. (2015). Acupuncture for functional dyspepsia: a single blinded, randomized, controlled trial. *Evidence-Based Complementary and Alternative Medicine*.

[14] Drossman D. A. (2006). The functional gastrointestinal disorders and the Rome III process. *Gastroenterology*.

[15] WHO Regional Office for the Western Pacific (2008). *WHO Standard Acupuncture Point Locations in the Western Pacific Region*.

[16] Zhou K., Fang J., Wang X. (2011). Characterization of De Qi with electroacupuncture at acupoints with different properties. *Journal of Alternative and Complementary Medicine*.

[17] Jenkinson M., Bannister P., Brady M., Smith S. (2002). Improved optimization for the robust and accurate linear registration and motion correction of brain images. *NeuroImage*.

[18] Smith S. M., Jenkinson M., Woolrich M. W. (2004). Advances in functional and structural MR image analysis and implementation as FSL. *NeuroImage*.

[19] Satterthwaite T. D., Elliott M. A., Gerraty R. T. (2013). An improved framework for confound regression and filtering for control of motion artifact in the preprocessing of resting-state functional connectivity data. *NeuroImage*.

[20] Yan C.-G., Cheung B., Kelly C. (2013). A comprehensive assessment of regional variation in the impact of head micromovements on functional connectomics. *NeuroImage*.

[21] van Dijk K. R. A., Sabuncu M. R., Buckner R. L. (2012). The influence of head motion on intrinsic functional connectivity MRI. *NeuroImage*.

[22] Biswal B., Yetkin F. Z., Haughton V. M., Hyde J. S. (1995). Functional connectivity in the motor cortex of resting human brain using echo-planar MRI. *Magnetic Resonance in Medicine*.

[23] Wang D., Buckner R. L., Liu H. (2014). Functional specialization in the human brain estimated by intrinsic hemispheric interaction. *The Journal of Neuroscience*.

[24] Fang J., Jin Z., Wang Y. (2009). The salient characteristics of the central effects of acupuncture needling: limbic-paralimbic-neocortical network modulation. *Human Brain Mapping*.

[25] Fang J., Wang X., Liu H. (2012). The limbic-prefrontal network modulated by electroacupuncture at CV4 and CV12. *Evidence-Based Complementary and Alternative Medicine*.

[26] Talley N. J., Haque M., Wyeth J. W. (1999). Development of a new dyspepsia impact scale: the Nepean Dyspepsia Index. *Alimentary Pharmacology & Therapeutics*.

[27] Tian X.-P., Li Y., Liang F.-R. (2009). Translation and validation of the Nepean Dyspepsia Index for functional dyspepsia in China. *World Journal of Gastroenterology*.

[28] Li L., Wang H. M., Shen Y. (2003). Chinese SF-36 Health Survey: translation, cultural adaptation, validation, and normalization. *Journal of Epidemiology and Community Health*.

[29] Biggs J. T., Wylie L. T., Ziegler V. E. (1978). Validity of the zung self rating depression scale. *British Journal of Psychiatry*.

[30] Liu X. C., Oda S., Peng X., Asai K. (1997). Life events and anxiety in Chinese medical students. *Social Psychiatry and Psychiatric Epidemiology*.

[31] Liu P., Qin W., Wang J. (2013). Identifying neural patterns of functional dyspepsia using multivariate pattern analysis: a resting-state fMRI study. *PLoS ONE*.

[32] Lima F. A. D. R., Ferreira L. E. V. V. D. C., Pace F. H. D. L. (2013). Acupuncture effectiveness as a complementary therapy in functional dyspepsia patients. *Arquivos de Gastroenterologia*.

[33] Chang X. R., Yan J., Lin Y. P., Yi S. X., Deng Y. J. (2001). Influence of puncturing tsusanli point on the gastrointestinal hormone of the plasma of the patients with functional dyspepsia. *Chinese Journal of Integrated Traditional and Western Medicine on Digestion*.

[34] Zhang D. H. (2013). *To study the influence of Shu-Mu-point combination on plasma GAS and GHR in Functional Dyspepsia patients [M.S. thesis]*.

[35] Drossman D. A., Ringel Y., Vogt B. A. (2003). Alterations of brain activity associated with resolution of emotional distress and pain in a case of severe irritable bowel syndrome. *Gastroenterology*.

[36] Jonsson B. H., Uvnäs-Moberg K., Theorell T., Gotthard R. (1998). Gastrin, cholecystokinin, and somatostatin in a laboratory experiment of patients with functional dyspepsia. *Psychosomatic Medicine*.

[37] Natale G., Pasquali L., Paparelli A., Fornai F. (2011). Parallel manifestations of neuropathologies in the enteric and central nervous systems. *Neurogastroenterology and Motility*.

[38] Buckner R. L. (2013). The brain's default network: origins and implications for the study of psychosis. *Dialogues in Clinical Neuroscience*.

